# Transient effects of tumor location on the functional architecture at rest in glioblastoma patients: three longitudinal case studies

**DOI:** 10.1186/s13014-016-0683-x

**Published:** 2016-08-17

**Authors:** Noora Tuovinen, Francesco de Pasquale, Massimo Caulo, Chiara Falletta Caravasso, Emilia Giudice, Roberto Miceli, Gianluca Ingrosso, Anne Laprie, Riccardo Santoni, Umberto Sabatini

**Affiliations:** 1 Department of Radiology, Santa Lucia Foundation, Rome, Italy; 2 Department of Neuroscience and Imaging, “G. D’Annunzio” University, Via dei Vestini 31, 66100 Chieti, CH Italy; 3 Department of Neurology, Medical University of Innsbruck, Innsbruck, Austria; 4 Faculty of Veterinary Medicine, University of Teramo, Teramo, Italy; 5 Department of Diagnostic Imaging, Molecular Imaging, Interventional Radiology and Radiotherapy, Tor Vergata University General Hospital, Rome, Italy; 6 Institut Claudius Regaud, Toulouse, France; 7 Department of Neuroradiology, University of Magna Graecia, Catanzaro, Italy

**Keywords:** Resting-state fMRI, Brain tumor, Radiotherapy, Functional connectivity

## Abstract

**Background:**

The cognitive function of brain tumor patients is affected during the treatment. There is evidence that gliomas and surgery alter the functional brain connectivity but studies on the longitudinal effects are lacking.

**Methods:**

We acquired longitudinal (pre- and post-radiotherapy) resting-state functional magnetic resonance imaging on three selected glioblastoma patients. These cases were selected to study three models: a lesion involving a functional hub within a central system, a lesion involving a peripheral node within a central system and a lesion involving a peripheral node of a non-central system.

**Results:**

We found that, as expected, the tumor lesion affects connections in close vicinity, but when the lesion relates to a functional hub, these changes involve long-range connections leading to diverse connectivity profiles pre- and post-radiotherapy. In particular, a global but temporary improvement in the post-radiotherapy connectivity was obtained when treating a lesion close to a network hub, such as the posterior Cingulate Cortex.

**Conclusions:**

This suggests that this node re-establishes communication to nodes further away in the network. Eventually, these observed effects seem to be transient and on the long-term the tumor burden leads to an overall decline of connectivity following the course of the pathology. Furthermore, we obtained that the link between hubs, such as the Supplementary Motor Area and posterior Cingulate Cortex represents an important backbone by means of which within and across network communication is handled: the disruption of this connection seems to imply a strong decrease in the overall connectivity.

## Background

Gliomas are the most common primary neoplasms of the brain [1] and based on *World Health Organization (WHO)* criteria they can be divided into low (II) and high (III and IV) grade tumors [2]. In particular, *glioblastoma multiforme (GBM)* is a malignant tumor (grade IV) whose patients have a poor median survival from 9 to 14 months. These high-grade gliomas are generally treated with surgery which is followed by adjuvant radiation and chemotherapy. In addition, patients might receive anti-epileptic drugs and corticosteroids. These treatments influence, e.g., in terms of fatigue, pain and sleep disturbance the cognitive performance of the patient [3]. Such performance is expected to depend both on the proximity of the tumor lesion to the eloquent brain areas involved as well as their resilient architecture of connectivity. In this respect, *functional magnetic resonance imaging (fMRI)* involving a determined cognitive task enables to assess the distance between the tumor and eloquent activation areas such as Rolandic or speech cortex [4], and to evaluate possible long-term deficits of the patient. Identifying a safe distance to functional areas allows a broader tumor resection which has proven to improve patient prognosis and quality-of-life. Thus, to localize functional activations remains essential since inter-patient differences exist and expanding tumors might shift structures or the brain might employ alternative locations through plasticity. However, task-fMRI highly relies on the patient compliance which is often severely limited, i.e., to perform a given task may result too demanding.

To overcome such a limitation, *resting-state fMRI (rs-fMRI)* can be performed on these patients to identify functional brain networks in the absence of a task. This technique provides access to an array of different cognitive domains. This is achieved by estimating connectivity maps either through seed-based [5] or *Independent Component Analysis (ICA)*-based approaches. While in seed-based technique prior knowledge on the functional location is needed, with ICA such information is not needed, and thus this approach allows to account for the possible shift of the expected structural and functional areas induced by the lesion. The estimation of resting-state networks (RSNs) is fundamental, not just to assess their internal coupling, but more importantly to estimate their cross-connectivity which relates to the efficiency of integration among the corresponding functional domains and their corresponding RSNs such as the internal cognition (*Default-Mode Network DMN),* attention (*Dorsal Attention Network DAN*) and executive functions (*SomatoMotor Network SMN*). These networks are well reported in the literature and their integration has been linked to the patients clinical profile [6]. For this reason, in this work we will focus on these systems among which the DMN seems to play a fundamental role as a connectivity core. The main DMN nodes are the M*edial Prefrontal Cortex (mPFC)*, *posterior Cingulate Cortex (*pCC) and left and right Angular Gyri. This network has been related to a state of internal cognition when the brain is not actively engaged in processing external stimuli [7]. The SMN is responsible for motor control and coordination and comprises functional nodes in right and left Central Sulci, as well as left and right *Supplementary Motor Area (SMA)*. The DAN that mediates conscious perception during tasks through top-down or goal-oriented processing [8] comprises dorsolateral prefrontal cortex, left and right Posterior Intra Parietal Sulci and the frontal eye fields. Now, since the integration between these networks has been shown to be realized through specific functional hubs, such as pCC [9–12], studying how they are affected in the presence of a glioma, may help to understand the altered functional communication in these patients [13]. Moreover, the longitudinal investigation of these aspects may address the eventual plasticity of the functional connectivity and its relationship with the patient clinical profile.

Typically, the integration of fMRI, in terms of activations or RSNs, into *radiotherapy (RT)* treatment planning is not done and this literature is limited [14–16]. Furthermore, there is a lack of literature on the longitudinal effects after the surgery and during RT [17]. Thus, it is still unclear, how the treatment affects the communication between different brain regions. However, the importance of this topic has increased in consequence of more precise RT treatments which allow better sparing of critical organs and boosting of dose to the tumor [18]. In this way, life expectancies of patients have prolonged and maintaining quality-of-life and cognitive performance is gaining more interest [19, 20]. A few previous studies addressed the connectivity in the above mentioned networks on these patients and they can be summarized as follows. It has been reported that the tumor itself reduces the connectivity of brain networks. In particular, Xu et al. [21] reported that the functional connectivity in low grade gliomas shows disturbed small-world manner and reduced global efficiency. Recently, Maesawa et al. [22] found in left hemisphere glioma patients (grades II-IV) a significant decrease in functional connectivity in the right Angular Gyrus of DMN and in the dorsolateral prefrontal cortex of the left *executive control network (ECN)*, whereas a significant increase in the right ECN was observed in the right parietal lobe. In addition, Niu et al. [23] found significantly reduced inter-hemispheric functional connectivity between primary motor cortices in patients (grades II-III). Furthermore, reduced DMN [24] and language network [25] connectivity with relation to the grade of the tumor has been detected. Mickevicius et al. [26] studied longitudinal changes caused by whole brain RT in a single patient being treated for brain metastases. They noted an initial decrease in functional connectivity followed by an increase in the intra-network correlation suggesting some level of functional recovery. In addition, other imaging techniques, such as MEG, reported evidence of deficits occurring at long-range connections [27–29].

However, these studies have not addressed the longitudinal effects caused by the lesion location after surgery and during RT. In this scenario, our work contributes to shed some light on the longitudinal effects during RT on the functional connectivity in these patients. Specifically, we investigated how primary brain tumors and their treatment with surgery and RT affect functional connectivity longitudinally. To this aim, we selected three case studies on GBM patients followed longitudinally before and after (pre- and post-) RT. As the aim is to assess how the treatment affects the functional architecture of segregation and integration in relation to the location of the tumor lesion, we present cases whose pathology involves a hub within a central network (pCC – DMN), a peripheral node of the same central network (mPFC – DMN) and a node belonging to a non-central system (preCG – SMN). In particular, a differential role is expected depending on the functional centrality of the nodes involved with the pathology. Our approach consists of different steps. First, ICA is used for network identification. Second, functional hubs are extracted from individual RSNs. Third, seed-based connectivity maps are studied for topography changes at different time points. Eventually, cross-connectivity matrices between nine selected hub locations from DMN, DAN and SMN are computed to study the within and across network communication.

## Materials and methods

### Subjects

A sample of 5 healthy subjects (mean age 32.80 ± 5.98 years, 5 males) was recruited to validate the proposed approach before conducting the analysis on 3 patients (mean age 61.67 ± 4.71 years, 3 females) pathologically confirmed with GBM. All participants provided written informed consent and the procedures were approved by the ethics committee at the Santa Lucia Foundation and were in accordance with the ethical standards of the 1964 Declaration of Helsinki. All GBM patients received three-dimensional conformal RT (3D-CRT). In addition, *Karnofsky performance score (KPS)* [30] and *Recursive partitioning analysis (RPA)*-classes [31] were evaluated pre- and post-RT.

### MRI data acquisition

fMRI data were acquired by means of a 3 T Philips Achieva (32-channel head coil) including anatomical sequences (T1, FLAIR, T1CE) and 3 five minute resting-state runs (FE EPI, TR/TE/flip angle = 2.00 *s*/30 *ms*/77°, field of view 224x224x106.4 *mm*, matrix size 2.8×2.8 *mm*, slice thickness 2.8 *mm*, SENSE factor). Subjects were told to lay still and relaxed and fixate a cross shown on the screen during the acquisition. Each resting-state run consisted of 180 volumes of axial slices in an interleaved order leading to 540 volumes acquired.

### Clinical history and neuroradiological description of the considered case studies

#### Case study 1: PAT_pCC

The first case study consists of a 55-year-old female whose lesion was in close proximity of posterior Cingulate Cortex and for this reason, in what follows we will label this patient as PAT_pCC. In this case, the KPS pre- and post-RT remained constant, namely 80 and the RPA-class was evaluated to be III. PAT_pCC underwent two MRI studies: the first one 2 months post-surgery and the second one 5 weeks post-RT. The time lag between these scans was 3 months.

In the first postoperative MRI study, T1 and FLAIR scans showed a resection of the neoplasm with a mixed signal cavity located in the left occipital-parietal lobes. Post-contrast T1-weighted scan revealed a thin and irregular enhancing ring. Maximum two dimension diameters of the cavity in the transverse plane were 56×24 *mm* and in plane area was 1230 *mm*^*2*^. A mural nodule that enhanced strongly and homogeneously after contrast injection was located in the anterior part of the cavity. A mild oedema surrounded the cavity with a mild effect on the left lateral ventricle.

In the second post-RT MRI study, the previous enhanced mural nodule showed an increase in size with a spread in the adjacent white matter and in the splenium of the corpus callosum indicating the growth of residual tumor. A T2-weighted extensive area of hyperintensity without post-contrast enhancement was located in the deep white matter of the left corona radiata and the centrum semiovale adjacent to the tumor, suggesting an early-delayed period of RT-related changes.

#### Case study 2: PAT_mPFC

The second patient considered consisted of a 65-year-old female with a lesion in close proximity to medial Prefrontal Cortex (thus labelled as PAT_mPFC). Her KPS pre- and post-RT remained constant in 90 and the RPA-class was evaluated to be IV. PAT_mPFC underwent three MRI studies: the first one 3 weeks post-surgery and the second one 4 weeks post-RT. The time lag between these scans was 3 months. The third scan was done 5 months post-RT.

In the first postoperative MRI study, T1 and FLAIR scans showed a resection of the neoplasm with a main low signal cavity located in the right frontal lobe. A focal and thin enhancing ring in the post-contrast T1-weighted scan was located in the posterior edge of the cavity. Maximum two dimension diameters of the cavity in the transverse plane were 30 × 18 *mm* and in plane area was 340 *mm*^*2*^. The surgical cavity was surrounded by a hyperintense T2-weighted area located in the adjacent frontal deep white matter. The right frontal horn of the lateral ventricle was slightly dilated.

In the second post-RT MRI study, an enhanced mural nodule was located post-contrast in the anterior and caudal edge of the cavity suggesting the growth of residual tumor. An increase in size of the previous T2-weighted hyperintense area without post-contrast enhancement was also observed. It was located in the adjacent frontal white matter, extending currently through the right corona radiata and the centrum semiovale.

In the third MRI study, the previous enhanced mural nodule increased in size associated to a diffuse and irregular enhancing ring of the cavity seen in the post-contrast scan. A further increase in size of the previous T2-weighted hyperintense area without post-contrast enhancement located in the adjacent frontal white matter, extending through the right corona radiata and the centrum semiovale was observed. The extension was in the rostrum and genu of the corpus callosum and in the white matter of the left frontal lobe. The right frontal horn of the lateral ventricle was still slightly dilated.

#### Case study 3: PAT_preCG

The third patient case presents a 65-year-old female with a lesion in proximity to *precentral gyrus (preCG,* this patient is labelled as PAT_preCG in what follows*)*. Her KPS decreased during the radiotherapy from 80 (pre-RT) to 70 (post-RT). RPA-class was evaluated to be V. In fact, during the first scan this patient was able to walk by herself, whereas during the last scan she used wheelchair and could walk small distance. This performance corresponds to lower KPS score of 50. PAT_preCG underwent three MRI studies: the first one 5 weeks post-surgery and the second one 5 weeks post-RT. Time between these scan points was 3 months. The third scan was done 4 months post-RT. This patient received chemotherapy between the 1st and 2nd scan points for 6 weeks.

In the first postoperative MRI study, the FLAIR scan showed a partial resection of the neoplasm with an area of hyperintense signal located in the right frontal lobe adjacent to the Rolandic fissure. A diffuse and irregular enhancing ring of the lesion was observed in the post-contrast T1-weighted scan with evidence of a fluid filled cavity located in the lateral side and a mural nodule located in the medial edge of the lesion. Maximum two dimension diameters of the lesion in the transverse plane were 30 × 24 *mm* and in plane area was 486 *mm*^*2*^. A mild oedema surrounded the lesion without effect on the right lateral ventricle.

In the second post-RT MRI study a reduction in the lesion size was observed. Maximum two dimension diameters of the lesion in the transverse plane were 20 × 18 *mm* and in plane area of 274 *mm*^*2*^. A diffuse, thick and irregular enhancing ring of the lesion was observed in the post-contrast T1-weighted scan. The peripheral oedema was reduced and the right lateral ventricle was slightly dilated.

In the third MRI study, the lesion showed a global increase in size and an intense, diffuse and irregular enhancing ring in the post-contrast T1-weighted scan. Maximum two dimensions diameters of the lesion in the transverse plane were 32 × 23 *mm and* in plane area was 630 *mm*^*2*^. The peripheral oedema was increased with reduction width of the adjacent subarachnoid spaces.

### Data preprocessing

The data preprocessing pipeline consists of several steps performed using a combination of tools including *dcm2nii* [32], *ITK-SNAP* [33], *the FMRIB’s Software Library (FSL)* [34] and custom homemade *MATLAB* scripts [35]. Semiautomatic tumor segmentations were done with ITK-SNAP based on anatomical images and reviewed by an expert radiologist. Preprocessing of the functional data consisted of excluding the first two volumes of each experimental run to allow the fMRI signal to reach steady state and scrubbing of the volumes with motion related artifacts. FEAT tool was used for preprocessing (motion and slice time correction, brain extraction, 5 *mm* smoothing, intensity normalization) and registration to T1-weighted image (6° of freedom) and 2 *mm* MNI152-template (12° of freedom) were based on FSL’s FLIRT and FNIRT.

### Resting-state network estimation

FSL’s MELODIC ICA was used to identify independent components over the three concatenated resting-state runs for each subject individually. Due to the disturbances in the networks of tumor patients, components closely resembling the typical topography of DMN, DAN and SMN were identified by visual inspection for each subject with taking into account possible variations induced by the pathology.

### Hub extraction

Based on the previous resting state literature [9, 10], the coordinates of 9 hubs corresponding to pCC, left and right Angular Gyri, left and right SMA, right and left Central Sulci, and left and right Posterior Intra Parietal Sulci were estimated. Due to the obtained differences in patients’ RSNs, also differences in hub anatomical locations were expected. Thus, we proceed as follows to identify hubs. We identified clusters from thresholded DMN, SMN and DAN. Then, we compared the hub coordinates reported in the literature to these clusters and if the hub coordinate fell within a cluster we assigned that hub to that cluster. Now within the assigned cluster, we defined as individual hub coordinates the location of the maximum IC weight within the selected cluster. This step was repeated for each of the nine clusters. In this way, the location of the functional hub could account for the eventual changes induced by the pathology. In addition, care was taken not to include hubs falling inside the tumor regions.

### Seed-based connectivity maps

In order to obtain the strength of connections within the DMN, seed-based connectivity maps where computed using pCC as a seed. In particular, time series for each voxel was extracted and the Pearson correlation coefficient was computed between each time series and the reference seed. This step was repeated for every run of every subject. Then, correlation values were Fisher-transformed according to *z = ln((1 + r)/(1-r))/2* and averaged across the three runs of every subject. Now, in order to obtain significant connections at the subject level, the connectivity values were thresholded corresponding to alpha = 0.05. We stress that this step allowed to identify individual thresholds, thus, accounting for the patients’ differences within the sample.

### Measure of network integration

Within and across network communication of DMN, DAN and SMN were studied by computing cross-connectivity matrices between the 9 selected hubs (pCC, left and right Angular Gyri, left and right SMA, left and right Central Sulci and left and right Posterior Intra Parietal Sulci) for each patient. Pearson correlation coefficient was computed between BOLD time series of each hub and results were averaged over three resting-state runs.

## Results

### Healthy subjects

To validate the proposed approach, our analysis pipeline was first applied to a sample of healthy subjects. The aim here is to show that the proposed approach replicates known findings on RSNs in healthy subjects. The results obtained on one representative healthy subject are reported in Appendix 1: Fig. 8. Among the RSNs identified by means of MELODIC (see Materials and Methods), three networks closely resembling the typical topography of SMN (Appendix 1: Fig. 8a), DMN (Appendix 1: Fig. 8b) and DAN (Appendix 1: Fig. 8c) in the literature are reported. It can be noted that the topographies of these RSNs, overlaid on the MNI-template, are in agreement with the literature [36]. In particular, the main nodes of these RSNs were identified: left and right SMA, left and right Central Sulci for SMN; pCC, left and right Angular Gyri and PFC for DMN; and left and right Posterior Intra Parietal Sulci for DAN.

As an additional check, connectivity maps were then computed by adopting SMA and pCC as seeds and the resulting significant connections (*p* < 0.05, FDR corrected) are reported (Appendix 2: Fig. 9a, b). The topographies of these systems are in line with the literature [37]. Moreover, 9 individual hubs from DMN, SMN and DAN were extracted (see Materials and Methods) and the cross-correlation matrix among these nodes was computed to investigate the within and across network communication (Appendix 2: Fig. 9c). The strong within network coupling (highlighted in black boxes) of this matrix supports the typical functional specialization of these RSNs. Moreover, some interesting across network interactions are identified such as the one between DMN and DAN nodes. Such connections support the expected functional integration of these systems.

### Patients

#### Within network coupling

**Fig. 1:**
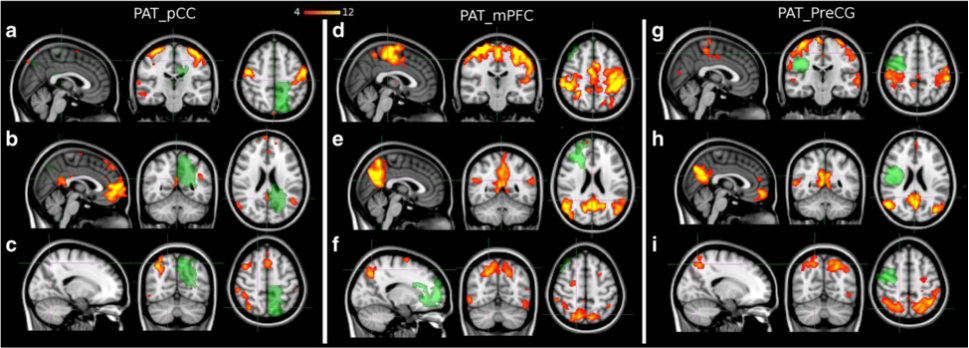
ICA-based RSNs pre-RT on three patients normalized to MNI. The Somatomotor (SMN) (**a**, **d**, **g**); the Default Mode (DMN) (**b**, **e**, **h**); and Dorsal Attention (DAN) (**c**, **f**, **i**) are reported with lesions (*green*). PAT_pCC has a lesion involving pCC, PAT_mPFC has a lesion involving mPFC, and PAT_preCG has a lesion involving PreCG

Here, results obtained on the selected patients are reported. Consistently with the results reported in healthy controls, the above three systems could be identified among the obtained ICs also in these patients (Fig. 1): SMN (Panel A, D, G), DMN (Panel B, E, H) and DAN (Panel C, F, I) pre-RT. Figure 1a-c reports the first patient case (PAT_pCC) with a lesion involving pCC, a functional connector hub of a central network (DMN). In Fig. 1d-f is shown the second patient case (PAT_mPFC) with a lesion involving mPFC, a local node of the DMN. Figure 1g-i relates to the third patient (PAT_preCG) reporting a lesion in right preCG outside the central system of DMN. In this figure, the location and the extension of the tumor lesion is overlaid in green. The tumor segmentation step is described in the Materials and Methods. In general, compared to healthy subjects, observed disruptions in the functional connectivity seem to strongly depend on the location of the tumor lesion.

From Fig. 1 it can be noted that in PAT_pCC the tumor lesion seems to have a global impact on the functional connectivity by affecting the topography of all three investigated networks. As a matter of fact, compared to the healthy RSNs, incomplete topology can be noted in them, i.e., SMA is absent from SMN, pCC is weakly coupled in the DMN and finally left Posterior Intra Parietal Sulcus is absent in the DAN. In PAT_mPFC and PAT_preCG instead, only local changes affecting the networks involved with the lesions are observed. In particular in PAT_mPFC, as expected, a local damage in DMN around mPFC can be noted, while both DAN and SMN are intact. In PAT_preCG, right Frontal Eye Fields connections are missing in the DAN while the DMN and SMN seem reasonably preserved.

Since these observations suggest a potential plasticity of the DMN, in what follows, we will focus on the longitudinal changes of this system. To this aim, the stability of DMN topology and in particular the seed-based connectivity maps (seed = pCC) longitudinally pre- and post-RT will be studied (see Materials and Methods). In particular, for PAT_pCC two sessions were acquired: at t1 (post-surgery) and t2 (5 weeks post-RT); while for the other patients an additional session could be acquired at t3 (4–5 months post-RT).

ICA DMN maps for PAT_pCC at t1 and t2 are reported in Appendix 3: Fig. 10. First, it can be noted that DMN could be identified at both time points by means of MELODIC ICA. Especially, the connectivity of pCC is affected by the close-by tumor lesion and is weaker than in healthy subjects at t1 (Appendix 3: Fig. 10a). In general, the overall DMN connectivity is weaker. In addition, the tumor has probably induced a dislocation of pCC ventrally and left Angular Gyrus anteriorly. Interestingly, post-RT (Appendix 3: Fig. 10b), an improvement of the network topology especially in the frontal node can be noted.

**Fig. 2:**
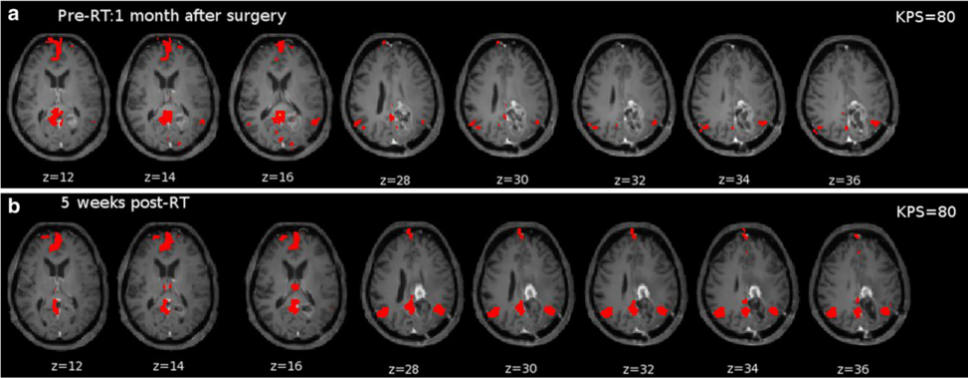
Seed-based connectivity maps for PAT_pCC overlaid on T1CE-weighted images normalized to MNI reporting significant connections (*p <* 0.05) from pCC. **a** pre-RT: connectivity to other DMN regions is weak. **b** 5 weeks post-RT: coupling to other DMN nodes is more focused. KPS at t1 and t2 is also reported showing stability

Now, to investigate the significant changes of DMN connectivity, Fig. 2 reports the seed-based connectivity maps from pCC at t1 (Fig. *2*a) and t2 (Fig. *2*b). It can be noted that DMN topography becomes more focused post-RT.

In Appendix 4: Fig. 11, ICA-based results for PAT_mPFC are reported. ICA DMN could be extracted at all three time points showing connectivity changes over time. At t1 (Appendix 4: Fig. 11a), a weakening of the mPFC can be noted but otherwise DMN topography seems intact. Post-RT connectivity at t2 (Appendix 4: Fig. 11b) becomes more focused and an improvement especially in the mPFC can be noted. At t3 (Appendix 4: Fig. 11c), a decrease of the connectivity to the frontal region suggests that the improvement accomplished was only transient since such connection is now lost again within the DMN. In addition, a general worsening of this network can be noted.

**Fig. 3:**
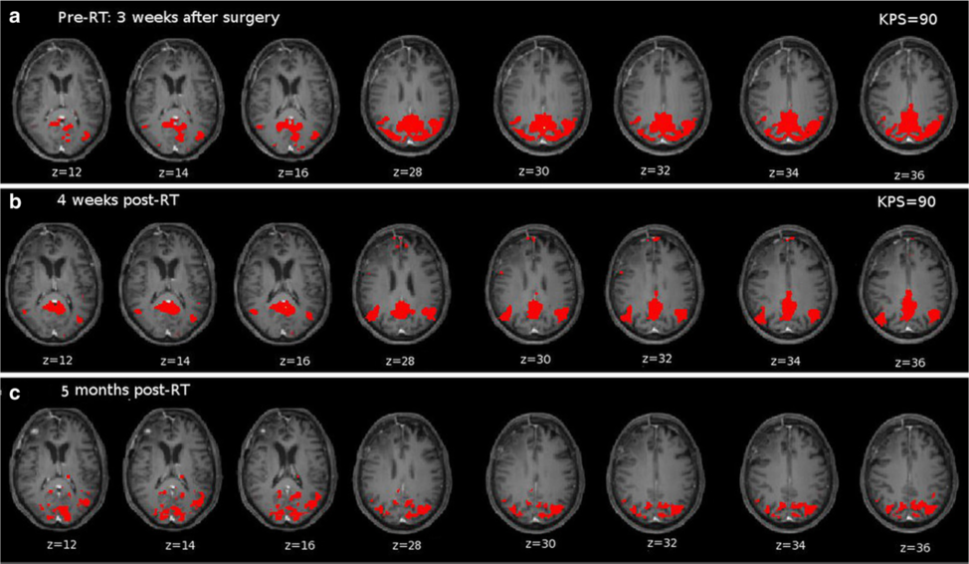
Seed-based connectivity maps for PAT_mPFC overlaid on T1CE-weighted images normalized to MNI reporting significant connections (p < 0.05) from pCC for **a** pre-RT: a spread of connectivity. **b** 4 weeks post-RT: connectivity improvement in frontal cortex. **c** 5 months post-RT: aspecific connectivity and no connection to frontal nodes. KPS at t1 and t2 is also reported

For the same patient, seed-based connectivity map from pCC at t1 is reported (Fig. 3a). A spread of connectivity can be noted likely suggesting a recovery mechanism following the surgery. A global connected area is noted involving the typical left and right Angular Gyri and pCC but such connections are not focused around the typically reported DMN nodes. At t2 (Fig. 3b), post-RT, pCC connectivity maps are more focused and frontal node connectivity is partially restored. Moreover, a clean topography without spurious connections can be noted. At t3 (Fig. 3c), the frontal node connectivity is lost again and the connectivity to other DMN regions is no longer obvious. There is an overall deterioration of the connectivity.

In Appendix 5: Fig. 12 we report the longitudinal results for the DMN from PAT_preCG characterized by a lesion far from DMN. It can be noted that at t1 (Appendix 5: Fig. 12a), the DMN includes all the expected regions and seems intact. At t2 (Appendix 5: Fig. 12b), post-RT connectivity in the right superior frontal node is lost and there is even a further loss of connectivity at t3 (Appendix 5: Fig. 12c).

**Fig. 4:**
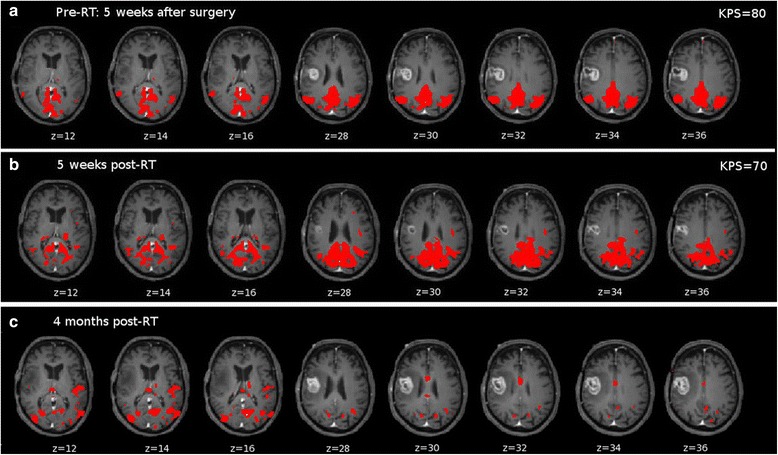
Seed-based connectivity maps for PAT_preCG overlaid on T1CE-weighted images normalized to MNI reporting significant connections (p < 0.05) from pCC. **a** Pre-RT: connectivity to frontal nodes of DMN is lost, **b** 5 weeks post-RT: spread of connectivity can be noted and **c** 4 months post-RT: typical pCC connectivity is lost. KPS at t1 and t2 is also reported

For the same patient, seed-based connectivity maps from pCC are reported (Fig. 4). Consistently with ICA-based results, also the seed-based connectivity shows an overall stability over time of this system. The difference is that ICs show typical DMN spatial pattern whereas looking at the connections from pCC, a disrupted network is noted. Thus, nodes are still connected but in particular from the DMN hub the connections are spread and aspecific. The connectivity from pCC deteriorates over time. This could probably be explained by the progression of the tumor. At t1 (Fig. 4a), the connectivity from pCC does not include frontal regions. At t2, post-RT (Fig. 4b), spread of connectivity from pCC can be noted. Furthermore, the frontal connections are missing. At t3 (Fig. 4c), connectivity from pCC becomes aspecific and no resemblance of connectivity to other DMN regions can be noted. For these patients, the acquired KPS at t1 and t2 are also reported (Figs. 2, 3 and 4).

So far, we have discussed the effect of the location of a lesion on functional connectivity. However, we cannot disentangle the combined effect of the lesion and the radiotherapy. In order to check the RT effects we report the dose delivered and its distribution in terms of isodose curves. As a control, in Table 1*,* the *Gross Tumor Volume (GTV)*, and volumes for functional hubs of pCC and SMA contours (*cm*^*3*^) together with their dose maxima, minima and mean (*cGy*) are reported. It can be noted, that the amount of dose delivered to nodes involved with the lesion is comparable, i.e., in terms of maximum and average dose delivered. This represents an important check since it rules out the hypothesis that the differences in the connectivity observed could be only due to the energy delivered in the GTV.

**Table 1:** Minima, maxima and mean doses received by Gross Tumor Volume (GTV), posterior Cingulate Cortex (pCC) and Supplementary Motor Area (SMA) for the three patients

Patient case	Contour	Volume (*cm* ^*3*^)	Dose_min_ (*cGy)*	Dose_max_ (*cGy)*	Dose_mean_ (*cGy)*	Age (years)	Tumor location	KPS (Pre)	KPS (Post)
PAT_pCC	GTV	41.9	5895.4	6252.8	6033.0	55	Intra-axial parieto-occipital	80	80
	pCC	3.5	5923.9	6019.1	5971.5				
	SMA	1.9	3576.0	6278.3	5581.4				
PAT_mPFC	GTV	6.2	6036.4	6290.0	6168.0	65	Frontal intra-parenchimal	90	90
	pCC	4.7	650.4	1478.9	1061.1				
	SMA	2.3	981.4	2445.7	1546.4				
PAT_preCG	GTV	8.4	5988.4	6255.2	6130.0	65	Prerolandic	80	70
	pCC	6.2	1040.0	3116.0	1518.4				
	SMA	1.7	1581.5	2615.8	2197.3				

As an additional control, the dose distribution maps (Appendix 6: Fig. 13) of these patients showing the isodose curves at 30, 50, 70 and 90 % of the maximum are reported. For PAT_pCC (Appendix 6: Fig. 13a), it can be noted that the dose has been maximized in the tumor and this posterior part of the brain is exposed to 50 % of the dose. As expected, being involved with the lesion, pCC is receiving 90 % of the dose. In Appendix 6: Fig. 13b, the isodose distribution for PAT_mPFC is reported. It can be noted that the dose has been maximized in the tumor situated in the frontal lobe. According to the percentage isodoses in this case, pCC is receiving less than 30 % of the planned dose whereas mPFC is receiving 50 % of the planned dose. In Appendix 6: Fig. 13c, the isodose distribution of PAT_preCG is reported. It can be noted that the dose has been maximized in the tumor near preCG. According to the isodoses, pCC is receiving between 30 to 50 % of the planned dose and mPFC is receiving less than 30 % of the planned dose.

#### Across network coupling

The previous results showed how these networks can be obtained in GBM patients and how they change over time. Since these systems are not segregated but central in the communication across the brain, we now investigate the across-network integration by extending our analysis from pCC to a sample of 9 known hubs involving DMN, SMN and DAN. As explained in Materials and Methods, this integration was estimated by means of a 9×9 cross-correlation matrix.

**Fig. 5:**
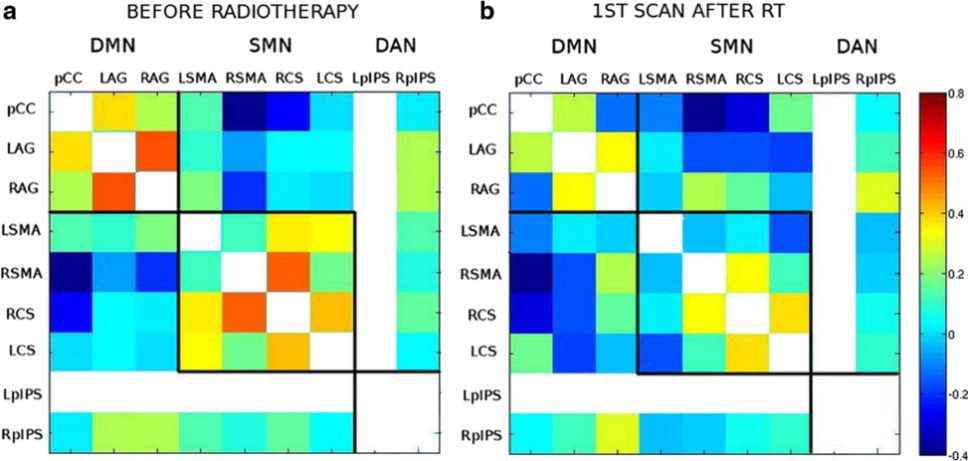
Cross-correlation matrices showing the interaction across 9 functional hubs (pCC, left and right Angular Gyri, left and right SMA, left and right Central Sulci, and left and right Posterior Intra Parietal Sulci) for PAT_pCC. **a** Pre-RT 1 month post-surgery: networks are acting in segregated manner and **b** 5 weeks post-RT: the overall communication between nodes is decreasing

Results for PAT_pCC are reported in Fig. 5. Please note that left Posterior Intra Parietal Sulcus was excluded since it was the only hub falling directly on tumor. Cross-connectivity matrix (Fig. 5a) shows strong coupling clustered around internal RSN nodes, hence, suggesting that DMN, SMN and DAN act in a segregated manner pre-RT. However, post-RT (Fig. 5b), an overall decrease, i.e., both within and across-connectivity can be noted. See for example pCC and left SMA interaction.

**Fig. 6:**
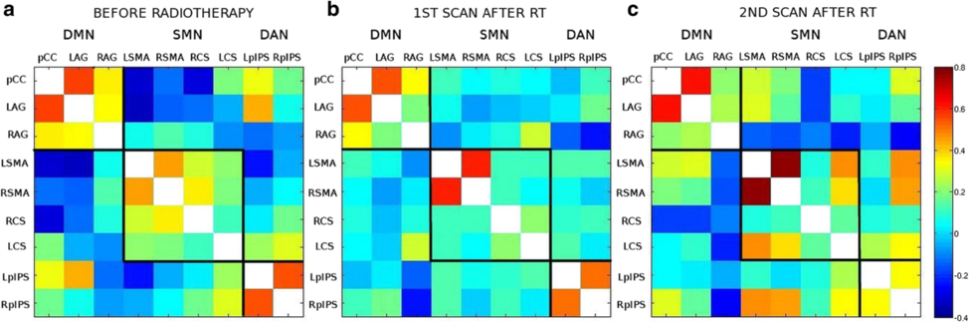
Cross-correlation matrices showing the interaction across 9 functional hubs for PAT_mPFC. **a** Pre-RT 3 weeks post-surgery: networks are segregated. **b** 4 weeks post-RT: flattening of the coupling can be noted and networks start to show more integrated behavior. **c** 5 months post-RT: the across-network communication increases even more

In Fig. 6a, in PAT_mPFC a similar segregated behaviour of all the networks is noted pre-RT. However, compared to the previous patient, at t2 post-RT (Fig. 6b) the coupling seems to flatten instead of decreasing and the communication across the networks increases. This might be due to the fact that the lesion has not involved important networks hubs, hence the connectivity between networks is maintained. Nevertheless, networks start to show more integrated behaviour as if part of the same system, i.e., the axis between SMA and pCC is preserved (this point will be elaborated in the Discussion). The connectivity across network hubs increases even more at t3 post-RT (Fig. 6c).

**Fig. 7:**
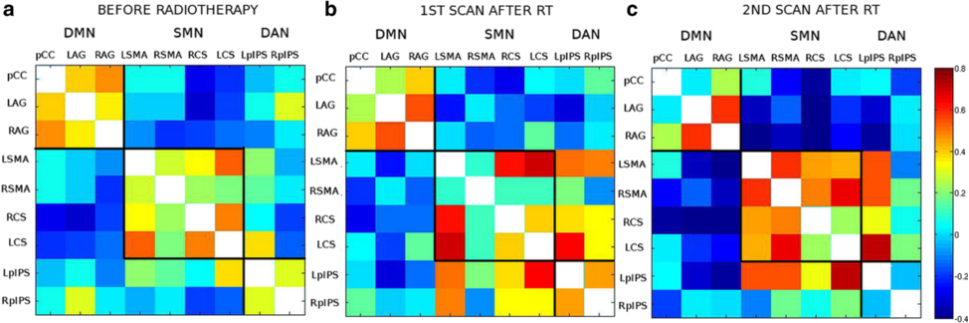
Cross-correlation matrices showing the interaction across 9 functional hubs for PAT_preCG. **a** Pre-RT 5 weeks post-surgery: networks show segregated behavior. **b** 5 weeks post-RT: DMN remains segregated. **c** 4 months post-RT: DMN remains segregated

**Fig. 8:**
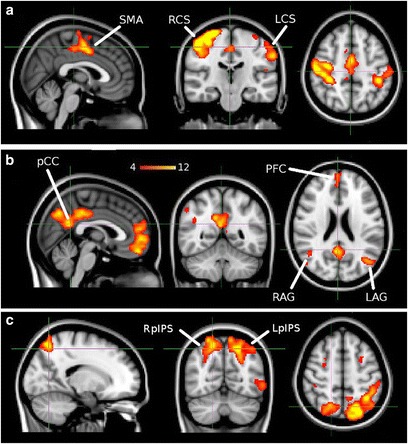
ICA-based RSNs in MNI-space for a representative healthy subject are in agreement with literature. **a** The Somatomotor (SMN), **b** Default Mode (DMN) and **c** Dorsal Attention (DAN) are reported with their typical nodes

**Fig. 9:**
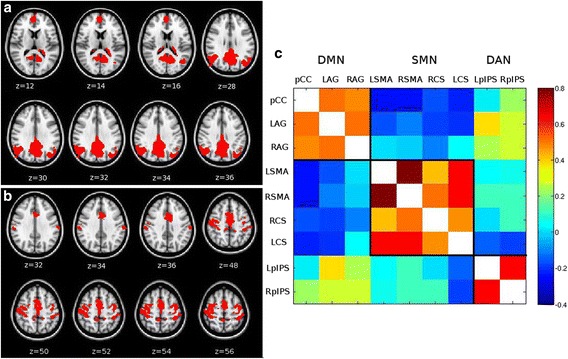
Seed-based connectivity maps for a healthy subject normalized to MNI. Maps show significant connections (p < 0.05) from **a** pCC and **b** SMA. **c** Cross-connectivity matrix for 9 known hubs from DMN, SMN and DAN

**Fig. 10:**
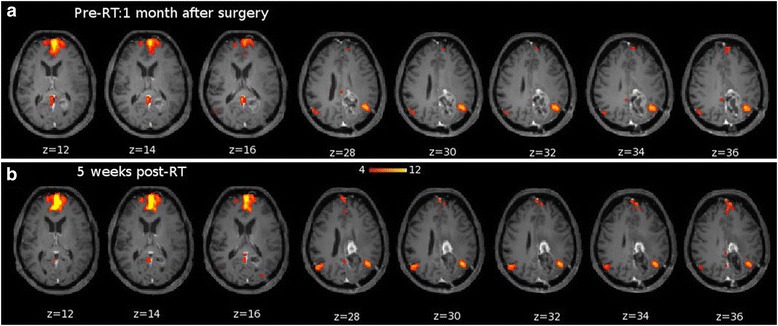
Default Mode Network topology for PAT_pCC overlaid on T1CE-weighted images normalized to MNI. **a** Pre-RT: tumor lesion is involving pCC and thus overall connectivity is weakened. **b** 5 weeks post-RT: improvement of prefrontal cortex connections

**Fig. 11:**
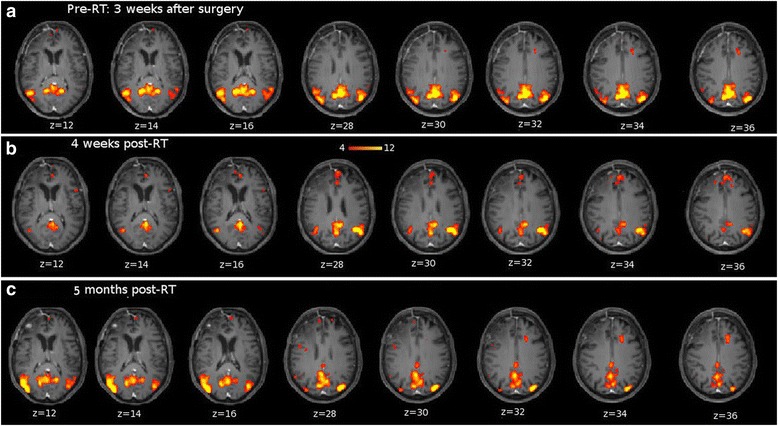
Default Mode Network topology for PAT_mPFC overlaid on T1CE-weighted images normalized to MNI-space. **a** Pre-RT: weakening of the medial prefrontal node. **b** 4 weeks post-RT: DMN is showing temporary improvement in the frontal node. **c** 5 months post-RT: topography to frontal regions is lost again

**Fig. 12:**
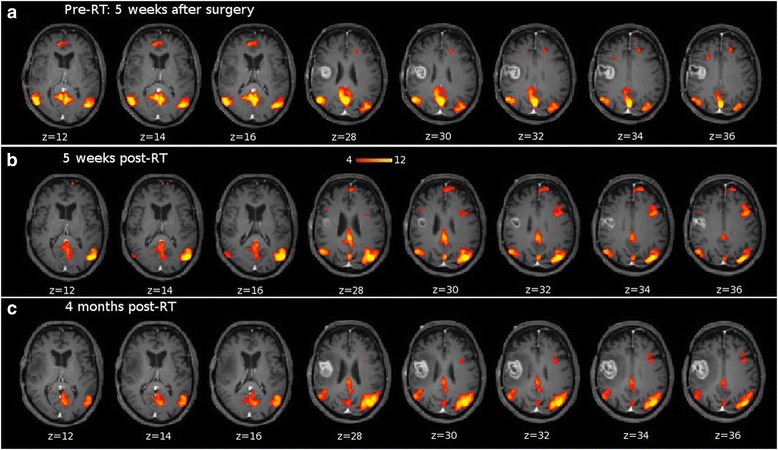
Default Mode Network topology for PAT_preCG overlaid on T1CE-weighted images normalized to MNI-space. **a** Pre-RT: DMN is intact. **b** 5 weeks post-RT: connectivity in right superior frontal node near tumor is lost. **c** 4 months post-RT: DMN topography to frontal region is lost

A similar functional segregation as above at t1, pre-RT, is noted for PAT_preCG (Fig. 7a). At t2 (Fig. 7b), SMN is showing stronger across network coupling. DMN remains segregated from the other two networks. At t3 (Fig. 7c), the across network interaction of SMN that we noted previously diminished. However, a stable segregation of the networks through t1, t2 and t3 can be noted.

## Discussion

### Overview

In the current study, we report on the functional connectivity changes after surgery and during RT on three selected glioblastoma case studies. The selection was based on our experimental hypothesis that a different impact on the structure of connectivity would be seen depending whether the pathology involved a hub within a central network (pCC – DMN), a peripheral node of the same central network (mPFC – DMN) or a node belonging to a non-central system (preCG – SMN). The developed methodology consisted of different steps. First, RSNs were extracted automatically by means of MELODIC. Second, within the obtained networks individual functional hubs were localized. This allowed to obtain specific hubs at subject level and at different time points so that eventual changes, i.e., compression or shift of functional areas, induced by the presence of the lesion could be taken into account. Third, we computed seed-based connectivity maps over time to estimate the plasticity of the systems under investigation in terms of changes in topography and intensity of coupling. Fourth, the integration across the DAN, DMN and SMN was estimated by computing cross-connectivity of nine extracted hubs within these RSNs. It must be stressed that due to the limited sample size, in this study we do not intend to draw any conclusion at the group level but these findings might pave the way for future studies on extensive sample of patients specifically selected whose enrolment is extremely demanding.

### Role of the proximity of lesion to hubs

The RSNs obtained with ICA, compared to healthy subjects, showed incomplete topographies involving some disrupted connections. These changes are in line with the hypothesis that the lesion vicinity to known nodes might cause anatomical and functional shift of the structures. These effects are supported by previous studies in glioma patients. In particular, Maesawa et al. [22] observed changes in connectivity in the contralateral side of left hemisphere tumor patients. This included significant decrease in functional connectivity in the right Angular Gyrus of DMN and in the dorsolateral prefrontal cortex of the left ECN, and a significant increase in the right ECN in the right parietal lobe. Furthermore, Esposito et al. [24] found a reorganization of DMN areas likely induced by tumor lesion in the left posterior parietal region. They found a reduced DMN integration of the left inferior parietal lobule and absence of connectivity with the right inferior parietal lobule. Furthermore, an increase of connectivity was present in the anterior-medial portion of the posterior cingulate. They noted that a fundamental difference between controls and tumor patients was a general reduced DMN connectivity within cortical areas and an increased connectivity with the hippocampus.

We note that disruptions of RSNs in tumor patients are highly dependent on the lesion location and thus the comparison with previous studies can be difficult. In particular, we hypothesized that the lesion proximity to known fundamental hubs would present a different effect on functional connectivity. To our knowledge, previous studies on functional connectivity on tumor patients have not addressed this effect. Based on our results, thus hypothesis was confirmed. As a matter of fact, we noted in PAT_pCC (see Fig. 1), that the proximity to pCC, an important functional hub, impacted the topography of the networks and the connectivity at a global level. Thus, changes were induced also on long-range connections, even on nodes that are far away from the tumor lesion. For example, SMA and left Posterior Intra Parietal Sulcus were disconnected within their own networks, namely SMN and DAN. This kind of global effect is in line with what is reported in previous MEG studies. As matter of fact, although not reflecting their results with functional hub locations, Bartolomei et al. [27] revealed that brain tumor patients had a loss of functional connectivity in comparison to controls which was not confined to the regions close to the tumor, but were widely spread in remote areas. Similarly, Guggisberg et al. [28] found scattered or diffuse areas with decreased connectivity confirming findings of Bartolomei et al. They also concluded that focal lesions produce a diffuse decrease in brain connectivity, when tumor patients are compared to a healthy population. Furthermore, Douw et al. [29] found decreased long-distance interhemispheric functional connectivity in the theta band after brain tumor resection. As a control, when we studied the connectivity changes induced in the patients with the lesion involving mPFC, a peripheral node of the DMN, we observed only a local damage limited to regions within DMN and around mPFC. Analogously, the lesion in preCG, outside the central system of DMN, affected locally the connections in the DAN (see Fig. 1).

### Differential longitudinal effects during radiotherapy on functional hubs

The role played by pCC as a functional hub seems even more important when we considered the longitudinal changes of connectivity induced by the lesion and during RT. To our knowledge, this is the first time that the post-surgery effects of the lesion during RT to functional connectivity on primary brain tumor patients are addressed.

Here, we noted that the DMN connectivity changes during the treatment. In particular, we observed a transient improvement in the overall connectivity of this network in two patients post-RT which seem to be highly dependent on the location of the lesion in regards of the functional nodes. Specifically, the combined effect of the surgery and RT on an important functional hub (pCC) helped to recover the connectivity globally even far away from the lesion (see Fig. 2). The observed global improvement might be due to the fact that the central node partly re-established its role of coordination across multiple networks [9–11]. Importantly, treating a location near mPFC does not seem to have an effect on other regions as this node is not acting as an important communication bridge, thereby the improvement is only noted locally (see Fig. 3). The improvement might be explained by the disappearance of oedema and inflammation effects in combination with the surgery and RT which together help to re-establish the typical topology of the networks.

As an important control, when the treatment did not involve nodes of DMN, no improvement in the network locally or globally was seen (see Fig. 4). On the contrary, there was a deterioration in the connectivity of this network which might also be explained by a faster growing tumor compared to the other patients.

**Fig. 13:**
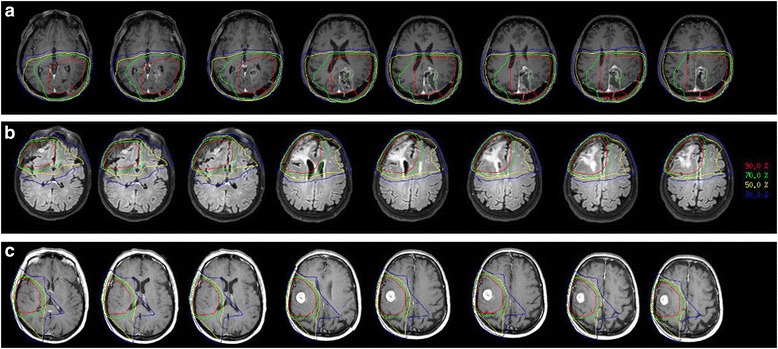
Percentage isodoses of 30 % (*blue*), 50 % (*yellow*), 70 % (*green*) and 90 % (*red*) of the maximum. **a** The dose for PAT_pCC has been maximized in the tumor in posterior part of the brain. **b** For PAT_mPFC the dose has been maximized in the tumor situated in the frontal lobe. **c** The dose for PAT_preCG has been maximized in the tumor near preCG

Although a lot of the functional changes seen during the RT might be explained by the relation of the surgical lesion site to the functional nodes, possible confounding effect is the amount of dose delivered to these patients as the dose distributions in the brain were different. To rule out this hypothesis we reported in Table 1 the average amount of dose delivered to these nodes and in Appendix 6: Fig. 13 the isodose curve distributions. It can be noted that all the patients received similar amount of dose but with distinct spatial distribution.

For two of the considered patients a third scan could be acquired as a follow-up after 4 months post-RT. Here we observed an overall worsening of the DMN connectivity. Hence, the possible improvement noted right after the RT seems to be only transient. These connectivity losses might be due to further spread and recurrence of the tumor. Furthermore, taking into account the severity of this kind of brain tumors, the deterioration of the functional connectivity is expected as the burden of the lesion is heavy and life expectancies of these patients are short.

### Longitudinal effect of the lesion on the functional segregation and integration of RSNs

Previous findings support the idea that when a functional hub is involved by a pathology, the recovery induced seems to be more global, i.e., affecting long-range connections within the same network where the original hub belongs. Now, this opens a new interesting questions on how the overall structure of functional connectivity involving different networks, and not just one single system, is affected by a lesion and how the recovery can be induced through a functional hub. To this aim, we investigated the cross-connectivity of nine functional hubs typically reported in the literature belonging to three of the main RSNs: the DMN, DAN and SMN. Although we were still able to recognize DMN, DAN and SMN post-RT, it seems that their communication is somehow altered. In healthy subjects it is known that these networks present a level of functional specialization, representing a strong internal connectivity and some level of across-network integration, represented by strong interaction with parts of the other networks. Previous studies have shown that with the absence of pathology, brain is structured to network communities which are connected by hubs. These communities act functionally segregated and specialized, while the hubs make sure that the information and communication across the networks communities is integrated [9–11, 38, 39].

We note differences in the segregation of networks in tumor patients compared to healthy subjects and this could be explained by the lesion involving specific functional hubs which then lose their ability to correctly maintain the information flow between the networks. In all of our patients, a segregation of networks in the similar way as in the healthy subject was noted pre-RT. Thus, the typical functional communication in the brain is still preserved at least to some degree in the presence of postoperative lesion. However, post-RT, different patterns of altered network communication were observed. The communication differences between patients might be explained by the diverse lesion locations which affect networks in separate ways.

In the case of tumor lesion close to pCC, both within and across network communication decrease (see Fig. 5). Since the tumor is also in the vicinity of other network hub (SMA), the brain seems to lose the link between these two functional hubs and this might explain why the overall communication decreases. This is in line with previous results on healthy subjects where the fundamental role played by the interaction between pCC and SMA was reported [39]. When this important functional backbone including pCC and SMA is lost this impacts severely the rest of the cross-network interactions.

The patient whose lesion is close to mPFC (but not SMA) also experiences changes in the way the networks communicate (see Fig. 6). However, in this case the communication across the networks seems to increase and more integrated behaviour appears as if part of the same system. In this case, the tumor has not involved network hubs, thus the connectivity between networks is preserved. However, networks start to show increasingly integrated behaviour since the axis between SMA and pCC is still fine and, thus these hubs are able to still communicate with each other. This observed spread of connectivity is in line with report mechanisms of recovery where functional specialization is somehow lost to privilege a more aspecific integrated functional structure.

As far as it concerns the patient whose lesion is far from DMN (see Fig. 7), we note that the brain is able to maintain some kind of functional specialization of networks, especially in the case of DMN as the tumor does not involve the important hub of this network.

To conclude, we noted that also the across network communication is altered in these patients and changes over time. The mechanism of these alterations seems to be dependent on whether the lesion has involved a central or a non-central functional hub. In particular, when the communication link between the main hubs is preserved, the integration between the networks increase as if the RSNs were part of the same system. In turn, when this link between central hubs was involved by the tumor lesion, their ability to talk lowered leading to a strong decrease in the overall connectivity.

Support from previous studies on patient populations with altered network communication exists. As mentioned, Xu et al. [21] found disturbed small-world manner and reduced global efficiency induced by low grade gliomas. Furthermore, Mickevicius et al., [26] studied a single patient with brain metastases being treated with RT and noted an initial decrease in functional connectivity followed by an increase in the intra-network correlation suggesting functional recovery. In addition, Yu et al. [40] found impairment in the state-related characteristics of both functional integration and functional segregation brain networks in schizophrenia patients.

### Limitations

In the current study, we present functional connectivity results based on a very limited sample, namely three brain tumor patients. This limitation is somehow related to the fact that to prove our hypothesis we need GBM patients presenting lesions in specific locations which are not easy to enroll. More importantly, to perform a longitudinal study on these patients is very difficult and time consuming. Therefore, our future studies will focus on applying our approach on a larger sample of GBM patients to strengthen the current results and extend these findings to an extensive sample.

Another limitation of our study is that the decline or increase in functional connectivity can be a combined effect of several aspects; size and location of tumor, surgery, RT, medication, as where as the patient age and co-morbidities. In addition, brain function might be affected by other metabolic, psychologic and social factors that are hard to measure [3]. Such effects can be hard to disentangle. Eventually, it is also difficult to study long-term effects for high-grade tumor patients as the duration of survival is short.

In the future, it will be fundamental to relate this kind of functional connectivity changes to disturbances in white matter tracts disrupted by the surgery and demyelination induced by RT. As tumor treatments are moving towards personalization, the possible future clinical impact of this kind of individual approach compared to group studies becomes more important. Brain networks and functional areas investigated with fMRI might give an insight on how the cognitive aspects of the patients are developing during the treatment. Whether the integration of fMRI information into RT treatment planning could further improve patients’ prognosis is an interesting and open research question. It has already been shown feasible by Wang et al. [41] who reported no significant difference between the 3DCRT and IMRT plans in terms of dose homogeneity while in the same time maximizing the dose to the tumor and sparing functional regions. However, it is still premature to draw a final conclusion on the factors leading to alterations in the connectivity as many factors play a role. However, the avoidance of brain necrosis in functional regions is becoming more relevant, as this could further improve patients’ performance and thus quality-of-life. This work presents preliminary results in understanding some aspects of the functional connectivity among the network nodes during RT. However, prospective assessment of such findings in extensive clinical trials is needed to provide a complete scenario of the treatment outcome in malignant gliomas.

## Conclusions

To our knowledge, this is the first study that reports on the longitudinal changes of functional connectivity on brain tumor patients during RT. In summary, we noted that tumor lesions may induce a number of changes in how the RSNs are disrupted and how the communication in the brain is altered. These preliminary results show that, as expected, tumor lesion was disrupting especially the nodes which were in close vicinity but changes also in long-range connections were observed when the a functional hub was involved. Moreover, notable connectivity changes occurred between pre- and post-RT in all these patients. A temporary improvement post-RT in the network was seen when treated near one of the network nodes. The global improvement seems to be related to treating close to a network hub, such as pCC, helping to establish communication to nodes further away in the network. As an important control, a node that is not centrally important for network communication could help to establish connectivity only locally. Eventually, these observed effects seem to be transient and on the long-term the tumor burden is too much to bare. Connectivity loss is usually seen further along during the disease and the overall connectivity declines following the course of the pathology.

## Abbreviations

3D-CRT: Three-dimensional conformal radiotherapy
DAN: Dorsal attention network
DMN: Default-mode network
ECN: Executive control network
fMRI: Functional magnetic resonance imaging
FSL: FMRIB’s software library
GBM: Glioblastoma multiforme
GTV: Gross tumor volume
ICA: Independent component analysis
KPS: Karnofsky performance score
mPFC: Medial prefrontal cortex
pCC: Posterior cingulate cortex
preCG: preCentral Gyrus
RPA: Recursive partitioning analysis
rs-fMRI: resting-state fMRI
RSN: Resting-state networks
RT: Radiotherapy
SMA: Supplementary motor area
SMN: Somato motor network
WHO: World Health Organization
